# Case of a Diseased Kidney

**Published:** 1795

**Authors:** Henry Yates Carter

**Affiliations:** Surgeon at Kettley, Near Wellington, in Shropshire.


III. *Cafe of a difeafed Kidney. By the fame.*

A SEAMAN, forty years old, of a plethoric habit, applied to me at Port Royal, in Jamaica, in 1782, with complaints nearly as follow:

A conftant aching, and fometimes acute pain, about the region of the right kidney, attended with a numbnefs of that fide, and pricking pains along the urethra, particularly when he paffed his urine; frequent inclination to make water, fometimes without ability to void any, and never voiding it but in fmall quantity; the urine itfelf being high coloured, depofiting a gritty lateritious fediment, fmelling very ftrong, and forming a film on its furface, which approached to a yellow colour. He complained likewife of a fenfe of fulnefs and heat at the neck of the bladder and about the perinæum, and could get but little reft in any other than an horizontal pofture. He was coftive, and had frequent naufea.

As he had a full pulfe, ten ounces of blood were taken from the arm, and a purging draught was adminiftered; after which he took occafional dofes of a mixture, the principal ingredients of which were diuretic falt and tincture of opium.

In the courfe of two or three days his pain was much alleviated, but the difficulty with which he voided his urine ftill continued.

He now complained of frequent and painful erections, more efpecially when an inclination to make water came on; he had likewife profufe colliquative fweats, and was coftive.

Care was taken to obviate this difpofition to coftivecoſtiveneſs, by means of purgative medicines and clyſters. Opium was now more liberally adminiſtered, and recourſe was occaſionally had to the warm bath. This laſt produced a certain degree of eaſe while he remained in it, but the ſenſe of ſtricture about the neck of the bladder continued, and the quantity of urine he was able to void ſeemed every day to become leſs, ſo that at the end of a fortnight it was deemed neceſſary to make uſe of the catheter, as he was unable to paſs a ſingle drop of urine without it.

By means of this inſtrument, from four to ſix ounces of turbid urine were drawn off twice a day. He had now much fever, and the pain about the neck of the bladder was become very acute, and ſeemed to affect him ſpaſmodically, as well after as previouſly to the introduction of the catheter. He was likewiſe frequently ſeized with violent pain, which began in his ſhoulders, and proceeded along the right ſide to the hip.

About a month after the firſt uſe of the catheter, he complained of a pain in the urethra, near the ſeat of the proſtate gland, particularly when the inſtrument was paſſing; and at times the catheter feemed to meet with fome refiftance at that part.

From this circumftance, together with the continuance of the pain in that and the neighbouring parts, and the frequent difcharge of drops of a mucous confiftence from the urethra, we were inclined to think that the principal feat of the difeafe was in the proftate gland, (efpecially as no appearance of calculus had been obferved), when a frefh fet of fymptoms directed our attention more particularly to the right kidney.

Thefe fymptoms confifted in a pain about the region of that kidney which he had before fcarcely mentioned, but which now (about feven weeks after he firft made his complaints known) was, at times, very fevere. His fhoulders alfo, but particularly the right, were fore, and at intervals acutely painful; the inguinal and axillary glands became fwelled, and fore to the touch; and he complained frequently of a fenfe of coldnefs in the direction of the right ureter, which was fucceeded by a painful inclination to make water.

From thefe circumftances it was fufpected that the right kidney, if not the chief fource of the extraordinary fymptoms I have been defcribing, defcribing, had at leaft fuffered confiderably. He was therefore urged to recollect any external injury he might have received. After a little hefitation he informed us, that about a month previoufly to his firft applying for relief, he had received feveral violent blows from the end of a large rope acrofs his loins, which for fome time had given him confiderable uneafinefs. In the courfe of a few days, however, he faid, the pain had gone off, but had returned at intervals; and as he had fuffered much, at different times, from gravel, he had afcribed his prefent complaints to that caufe.

At the time he made known thefe particulars, he was in a very reduced condition; his ftomach was become fo extremely irritable, that it retained but little of what was given to him either of food or medicine; and about a week afterwards he died.

On diffection the urethra was found to be in a healthy ftate, but the proftate gland was a little enlarged. The bladder contained about eight ounces of turbid urine, mixed with a purulent fluid, very offenfive to the fmell. The right ureter was much enlarged, and filled with the fame kind of fœtid matter. The kidney ney on the fame fide was enlarged nearly to thrice its natural fize, and on being opened was found to be in a ftate of fuppuration, and to contain a confiderable quantity of fœtid pus, fo that the internal fubftance of the kidney was in a great meafure deftroyed.

There was no appearance of calculus; and the other kidney, as well as the reft of the abdominal vifcera, appeared to be in a natural ftate.

It may be doubted, perhaps, whether the affection of the kidney, in this cafe, ought folely to be attributed to the effects of the blows that were inflicted; but allowing the kidney to have been previoufly difeafed (and the complaints the patient had already experienced, and which he attributed to gravel, render it not improbable that it was fo); ftill there can, I think, be no doubt that the fuppurative procefs which took place was haftened, if not immediately occafioned, by external violence. And of fuppuration of the kidnies from external injury, in any refpect fimilar to the prefent, I have been able to meet with no example in books. Different fyftematic writers do indeed enumeenumerate external contuſion among the remote cauſes of nephritis, but I do not find, in any of them, an inſtance of ſuch an affection from ſuch a ſource; ſo that I flatter myſelf the caſe I have related will be thought worthy of being recorded.

It ſhows that a frequent inclination, without ability, to make water, is not always occaſioned by gravel or calculous concretions; and it affords a ſtriking inſtance of the influence an organ like the kidney may have upon parts not only contiguous to, but even remote from the ſeat of diſeaſe.

